# Dynamic regulation of small RNAs in anthocyanin accumulation during blueberry fruit maturation

**DOI:** 10.1038/s41598-021-93141-8

**Published:** 2021-07-23

**Authors:** Xiaobai Li, Yan Hong, Aaron Jackson, Fangqi Guo

**Affiliations:** 1 Zhejiang Academy of Agricultural Sciences, Hangzhou, 310021 China; 2 Zhejiang Sci-Tech University, Hangzhou, 310018 China; 3 Unaffiliated, South Oak, Stuttgart, AR 72160 USA

**Keywords:** miRNAs, Gene expression

## Abstract

Blueberry is rich in anthocyanins which accumulate during fruit maturation. Previous studies mostly focus on their translational/transcriptional regulation, but usually underestimate their post-transcriptional regulation, e.g. small RNAs. This study aimed to identify sRNAs and their potential pathways associated with anthocyanin biosynthesis. During three typical phases of fruit maturation (green, pink, and blue), we investigated dynamic changes of sRNA by deep sequencing sRNA and examined the interaction of sRNAs with their target genes by degradome and RLM-PCR. During maturation, up-regulation of VcmiRNA156 and VcmiR393 resulted in down-regulation of VcSPLs and VcTIR1/AFBs, respectively. An important gene of anthocyanin biosynthesis, VcDFR, was substantially down-regulated at both the mRNA and protein levels, and potentially responded to regulation of VcSPLs and VcTIR1/AFBs. Additionally, indole acetic acid (IAA) and abscisic acid (ABA) were involved in the regulation of anthocyanin biosynthesis by interacting with VcmiR393-TIR1/AFBs and VcmiRNA319-VcMYBs respectively. This information provides another insight into blueberry anthocyanin biosynthesis.

## Introduction

Blueberry is a very desirable and nutritious fruit, which is well known for its richness in anthocyanins. Anthocyanins are primarily found in its ripe fruit and accumulate in fruit pericarp. Antioxidant properties of blueberry for health benefits are largely due to anthocyanins, which are linked to prevention of macular degeneration, anticancer activity, age-related cognitive decline^1,2^, and reduced risk of heart disease^3^. The fruit maturation is characterized by the changes of its organoleptic properties and phytonutrient composition. During maturation, a substantial increase of anthocyanin is responsible for the changes from green to pink, and then to dark blue. The increased anthocyanins have attracted much attention to the study of mechanism underlying their biosynthesis from different perspectives e.g. gene and protein expression^4,5^.

In plants, anthocyanin precursors are formed through the general phenylpropanoid pathway and modified into anthocyanins through the downstream flavonoid biosynthetic pathway^4^. In the initial committed step of the phenylpropanoid pathway in higher plants, phenylalanine ammonialyase (PAL) catalyzes the conversion of L-phenylalanine into trans-cinnamate. Trans-Cinnamate 4-Monooxygenase (C4H) and 4-Coumarate-CoA Ligase (4CL) respectively catalyze the second and third steps of phenylpropanoid pathway. Flavonoid biosynthesis begins with chalcone synthase (CHS) working on the condensation of three molecules of malonyl CoA and one of p-coumaroyl CoA synthesized from phenylalanine. Then, naringenin 3-Dioxygenase (F3H), flavonoid 3'-Monooxygenase (F3’H), and flavonoid 3',5'-hydroxylase (F3′5’H) catalysis respectively result in cyanidin and delphinidin anthocyanidins. Anthocyanidin synthase (LDOX/ANS) transforms leucoanthocyanins into anthocyanidins. Further, anthocyanidins are glycosylated by uridine diphosphate (UDP)-glucose: flavonoid-O-glycosyl-transferase (UFGT). O-methyltransferases (OMTs) catalyze the formation of O-methylated anthocyanins such as malvidin, peonidin, and petunidin. The transcription of these structural genesis usually regulated by three types of transcription factors (TFs) together, i.e. DNA-binding R2R3 MYB, MYC-like basic helix–loop–helix (bHLH or MYC) and WD40-repeats^4,5^, Another subset of TFs, SQUAMOSA promoter-binding protein-like (SPL/SBP) family participate in regulation of these structural genes as well^6^.

Notably, miRNAs are also involved in anthocyanin biosynthesis through their target genes^7^. In Arabidopsis, miR828 negatively regulates anthocyanin biosynthesis by repressing transcriptional factors, MYB75, MYB90, and MYB113^8^, while miR156 positively regulates anthocyanin biosynthesis by suppressing a negative regulator SPL9^6^. In grape, miR828 and miR858 negatively regulate VvMYB114 to promote anthocyanin biosynthesis^9^. In tomato, miR858 merges into anthocyanin biosynthesis via controlling transcriptional factors, SlMYB7-like and SlMYB48^10^. Recently, a draft-genome^11^, and another haplotype-phased genome^12^ have been successively built. The draft-genome is based on RNA-Seq data derived from five fruit developmental phases of a southern highbush cultivar “O’Neal”. A number of potential blueberry miRNAs and their corresponding targets have been identified^13^. Of these, the miRNA involved in the transitions from flower, to white fruit and then to blue fruit have been characterized^14^.

In this study, we focused on anthocyanin accumulation during fruit maturation (green, pink and blue phases) from the perspective of miRNA regulation. The sRNA sequences from the three phases of fruit maturation were used to identify miRNAs while degradome and RLM-PCR were to identify miRNA cleavage sites. By analysis of miRNAs and their target gene expression as well as the interactions that occur between them, we revealed a piece of the miRNA network regulating anthocyanin accumulation in blueberry fruit.

## Materials and methods

### Plant material

**Figure 1:**
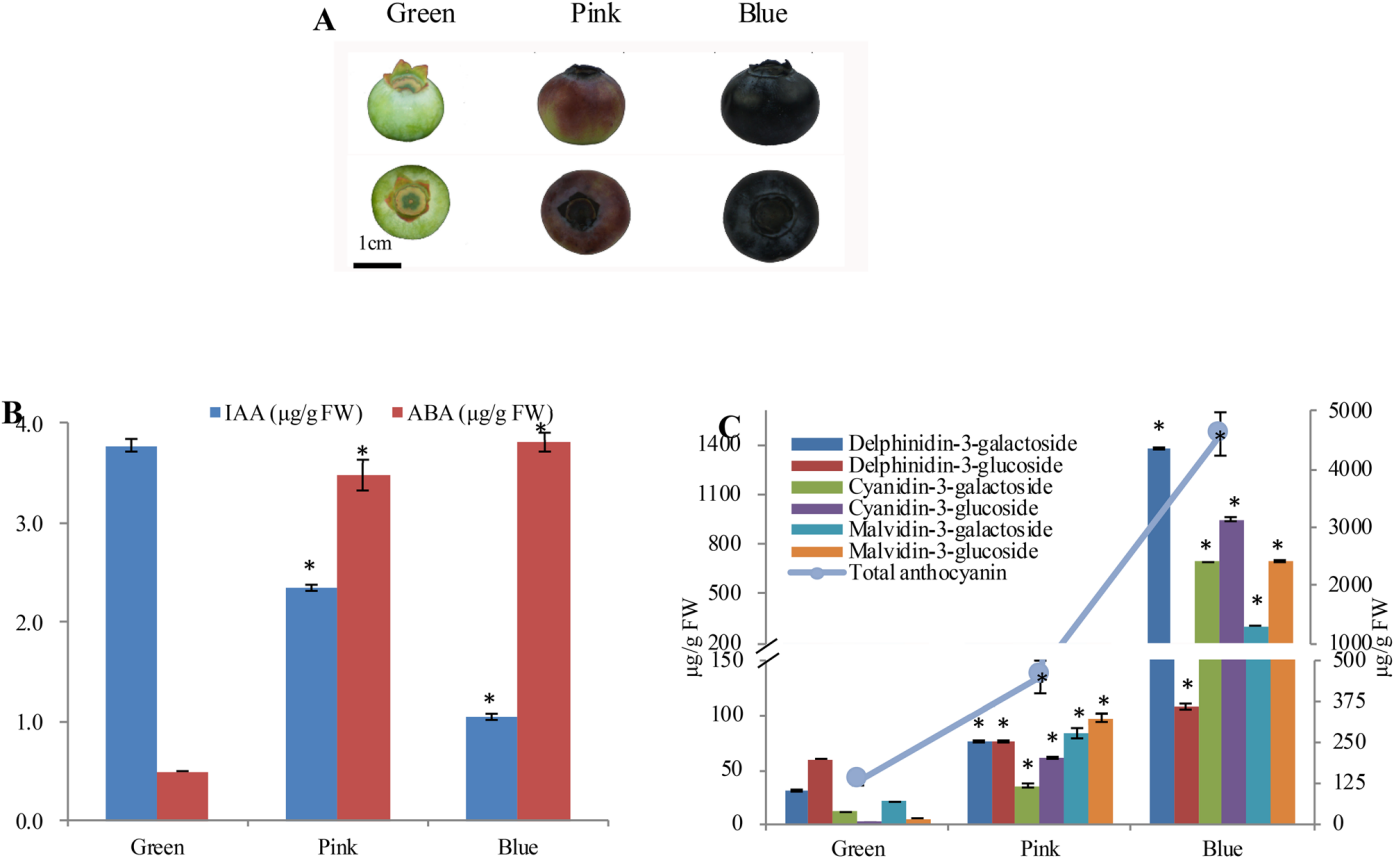
The dynamic changes of **(A)** fruit morphology **(B)** two kinds of hormones, and **(C)** total anthocyanin (AN) and six other major anthocyanins present during blueberry fruit maturation. Error bars represented standard errors from three biological replicates (ten fruit as one replicate). Measurement unit in **(A)** was in millimeters (mm).  "*" indicates differences between the green, pink and blue phases are significant (T test, *:P < 0.01).

Blueberry fruits were collected from three or more plants of the O’Neal variety of southern highbush blueberry (*V. corymbosum*) at green, pink and blue phases during the growing season (May, 2017) at Yangdu (North: 30.44; East: 120.41), Zhejiang. In the “green phase” fruits were green and fully rounded. The “pink phase” fruits consisted of berries which were partially pigmented but still firm. While the “blue phase” fruits were defined as fruits were soft and fully blue colored (Fig. 1A). Fruits were frozen in liquid nitrogen as they were harvested from the plant and then stored in a − 70 °C freezer. At least ten fruits were pooled as one replicate. Three biological replicates for each phase were used for all experiments. Whole fruit was ground in liquid nitrogen into powders, and subsequently used for further analysis.

Fruit samples were collected from the orchard of Zhejiang Academy of Agricultural Sciences, which had been approved by the local government. Identification of the plant was undertaken by Professor Ming Xie. Voucher specimens (ZAAS20160510) were kept at the Zhejiang Academy of Agricultural Sciences. Experimental research on plants complied with the guidelines of Zhejiang Academy of Agricultural Sciences. Field studies were approved by the Chinese government and implemented in compliance with the laws of the People’s Republic of China.

### Quantification of anthocyanin, IAA, ABA and gene/protein expression

Total anthocyanin content was determined using the pH differential method as described in Li et al.^4^. The content of del-3-glucoside, del-3-galactoside, pet-3-glucoside, pet-3-galactoside, mal-3-glucoside, and mal-3-galactoside was determined using the HPLC method as Li et al. (2019) described^4^.

Plant hormones indole acetic acid (IAA) and abscisic acid (ABA) were quantified by following these procedures: fruit samples were weighed, ground with extraction solvent (methanol:H2O:acetic acid = 80:20:1) and stored at 4 °C overnight. The resulting slurry was centrifuged at 8000*g* for 10 min. The supernatant was evaporated with nitrogen flow to water phase. Citric acid was added to the water phase of the supernatant until PH 2–3 was reached and then extracted with ethyl acetate three times. The supernatant was merged together, evaporated with nitrogen flow to dryness and methanol was added to bring the volume to 0.5 ml. The extract was passed through a 0.22 μm microporous membrane filter. The final filtrate was stored at – 20 °C until HPLC analysis. Chromatographic separation was performed in a RP-HPLC ACE C18 column through flow phase (methanol:water = 800:200) at a flow rate of 0.8 mL/min and 30 °C. The fluorescence detector was set as λ = 280 nm.

The expression of the genes/proteins mentioned in this study was quantified based on the transcriptomic (http://www.igbquickload.org/blueberry/) and proteomic data^4,11^. The transcriptomic and proteomic data was obtained from maturing fruits of the same highbush blueberry variety “O’Neal”.

### Small RNA library construction, sequencing and bioinformatic analysis

Total RNA was extracted from fruit tissues using Qiagen miRNeasy Mini Kit. The sRNA libraries were built using purified RNA and ligated with 3′ and 5′ adapters (Illumina, San Diego, CA, USA). The first strand of cDNA synthesis was synthesized by reverse transcription, and then the synthesized cDNAs were amplified by a PCR process of 15 cycles (each cycle: denaturation, annealing and extension of 10 s at 98 °C, 30 s at 60 °C and 15 s at 72 °C). The amplified products (about 140 bp) were purified by 6% polyacrylamide gel electrophoresis, and then sequenced by using Hiseq2000 (Illumina, San Diego, CA, USA), using the Illumina paired-end/single-end RNA-seq approach.

After filtering the adaptors and the reads with excessing small tags, the clean reads ranging 18–40 nt in length were processed for further bioinformatics analysis. The raw sequence data have been submitted to the NCBI Sequence Read Archive (SRA accession: PRJNA558093). Clean reads were aligned to the (ftp://ftp.ncbi.nlm.nih.gov/genomes/Solanum_lycopersicum/) reference genome using the tool Bowtie (http://bowtie.cbcb.umd.edu), and the reads mapped to multiple positions in the genome and to other non-coding RNAs (tRNA, rRNA, snRNA and snoRNA) were discarded. The remaining sequences which exactly matched the conserved miRNAs with miRbase (version 21, http://www.mirbase.org) were identified as conserved miRNAs and quantified by aligning reads to miRbase with a quantifier.pl from miRDeep2 package. Novel miRNAs were predicted by miREvo and mirdeep2 through exploring the secondary structure and identifying the Dicer cleavage site and the minimum free energy of the sRNA tags unannotated in the former steps.

The expression of miRNAs was normalized with transcripts per million (TPM, TPM = (read counts * 10^6^)/(total transcript counts)). miRNA differential expression analysis was performed based on a threshold of a twice fold change (or log2FC > 1) and P-value < 0.05. The target genes of the corresponding mature miRNAs were predicted from TargetScan, RNAhybrid and miRanda. Target genes were submitted to Gene ontology (GO) and Kyoto Encyclopedia of Genes and Genomes (KEGG) enrichment analyses^15^.

### Quantification of miRNA expression

miRNA expression levels were estimated by TPM (transcript per million), which is normalized by mapped readcount/total reads*1,000,000. Differential expression analysis of two floral development stages was performed using the DESeq R package (1.8.3)^16^. The threshold of significant differential expression was set as FDR < 0.05, |log2(foldchange)|> 1. The specific miRNAs with different expression were verified by stem-loop Real-time PCR (RT-qPCR) as described previously^17^. The miRNA was reverse-transcribed using specific stem-loop primers and their reverse-transcribed products were used as template for RT-qPCR with gene-specific primers (Table S1). The U6 snRNA was referred for normalization. All reactions were assayed in three biological and technical replications, and performed in an ABI PRISM 7900HT (Applied Biosystems, USA) using Platinum SYBR Green qPCR SuperMix-UDG (Invitrogen, USA). The PCR cycle was set for a pre-denaturation and hot start Taq activation at 95 °C for 5 min, then 40 cycles of 95 °C for 15 s, and 60 °C for 30 s.

### Degradome and RLM-RACE for cleavage site prediction and verification

RNA from green, pink and blue fruits were mixed equally. Approximately 20 μg mixed RNA were used to prepare a mixed degradome library. The method followed these procedures. (1) Approximately 150 ng of poly(A) + RNA was used as input RNA and annealed with Biotinylated Random Primers. (2) Strapavidin capture of RNA fragments through Biotinylated Random Primers (3) 5’ adaptor ligation to only those RNAs containing 5’-monophosphates. (4) Reverse transcription and PCR (5) Libraries were sequenced using the 5’ adapter only, resulting in the sequencing of the first 36 nucleotides of the inserts that represented the 5’ ends of the original RNAs. Single-end sequencing (36 bp) was then performed on an Illumina Hiseq2500 following the vendor’s recommended protocol. The purified cDNA library was sequenced on Illumina Hiseq 2500. Raw sequencing reads were obtained and submitted into NCBI (SRA accession: PRJNA558183). RNAseq reads were removed from adapter sequences, etc. and then mapped to the blueberry transcriptome. The mapped data were analyzed for alignments of sRNA/mRNA to identify the cleavage sites.

The 5’-end cleavage product of VcSPL12 (CUFF.8983.1 and CUFF.8983.2) and VcTIR1 (CUFF.41115.1) were determined by a RLM 5’-RACE using the RLM-RACE GeneRacer kit (Invitrogen, Carlsbad, CA, USA), as described in Li et al. described (2015)^17^. After reverse transcription, the 5’-end of the cleavage product was amplified using the GeneRace 5’ primers, gene specific reverse primers (Table S2) and their products were sequenced. qRT-PCR for SPLs and TIR1 was performed with primers (Table S3) in a LightCycler real-time PCR instrument (Roche, Switzerland), and SYBR FastStart, Essential DNA Green Master Mix (Roche, Switzerland). ACTIN (CF811156) was used as an internal control for targets.

## Results

### Anthocyanin accumulation and hormones change during maturation

During fruit maturation, color changes from full green, to pink and then to full blue (Fig. 1A). During the process, IAA was down-regulated, while ABA was up-regulated (Fig. 1B). The up/down-regulation of hormones mainly occurred in the pink/green. Anthocyanin content increased by 245.57% in pink/green, and soared by 921.91% in pink/blue (Fig. 1C). Six major kinds of anthocyanins i.e. del-3-glucoside, del-3-galactoside, pet-3-glucoside, pet-3-galactoside, mal-3-glucoside, and mal-3-galactoside were all up-regulated^4^. The increasing level of these anthocyanins was similar to what was seen in total anthocyanin accumulation. Collectively, anthocyanin increased in content as fruit matured, while two kinds of hormones showed different expression patterns.

### Identification of miRNAs, their putative targets, and functional analysis

Nine sRNA libraries were constructed, derived from green, pink, and blue fruit respectively. Deep sequencing generated 11,467,559.11 raw reads, and 573,377,955.56 nt per library. After removing adapter and poly-A tail, clean reads averaged 11,453,991.89 per library. The reads ranging from 18 to 40 nt were selected for subsequent analysis, resulting in 7,522,791.89 reads, and 166,817,649.78 nt per library. Among these reads ranging from 18–24 nt, sRNA ranging from 21 to 24 nt was common (potential miRNA), while the 24 nt RNA (potential siRNA) was the most abundant (Fig. S1). Approximately, 72.61% of 18–40 nt reads could be perfectly mapped to the blueberry draft-genome. The mapped sRNA sequences were annotated as several RNA classes (Table S4).

The mapped sRNA reads were searched against known miRNAs database. A total of 710 unique conserved sRNA representing 380,259 reads were identified (Table S5). Of these, 68 mature miRNAs corresponded to 60 hairpin precursors that adopted hairpin structures resembling the fold-back structure of a miRNA precursor. Additionally, 5,212 unique miRNAs representing 22,864 reads were derived from 161 novel mature miRNAs corresponding to 107 hairpin miRNA precursors. A total of 64 conserved and 334 novel miRNAs targeted 11,325 sites of 11,028 potential unigenes, with an average of 27.71 per unigenes/miRNA (Table S6). Here, 1,230 (11.15%) unigenes were targeted by mutiple miRNAs, while 303 (76.13%) miRNAs targeted multiple unigenes. Obviously, miRNA regulated multiple unigenes or multiple sites in a single unigene. In GO analysis, a large part of unigenes were involved in cellular and metabolic functions of biological process, and binding and catalytic activity of molecular function (Fig. S2). In KEGG, unigenes were enriched in glycine, serine and threonine metabolism, RNA degradation, zeatin biosynthesis, etc. These unigenes included MADS-box genes involved in flower and fruit development, such as B gene, Deficiens gene (DEF), and E gene Agamous-like, (AGL), and another non-MADS gene, *APETALA2* (*AP2*)*-LIKE*. Some were involved in biosynthesis pathway of flavonols, anthocyanins, and proanthocyanins, e.g. 4CL, CHI, CHS, F3'H, FLS, dihydroflavonol reductase (DFR), leucoanthocyanidin reductase (LAR), et al. Others were involved in hormone signaling e.g. ABA receptors, auxin response factors (ARFs), ethylene-responsive transcription factor (ERFs), jasmonate ZIM-domain protein (JAZ) et al.

One mixed degradome library of green, pink and blue fruits was constructed to identify miRNA cleavage sites. After filtering the low quality and repeat sequences, 13,114,294 clean reads and 823,072 unique clean reads were mapped to the blueberry fruit transcriptome. Finally, 12,423,888 (94.74%) mapped reads and 3,788,804 unique mapped reads (56.28%) were analyzed to identify candidate target genes and miRNAs, resulting in the 173 cleavage sites of 73 novel and 15 conserved miRNAs, e.g. VcmiR408b-3p to CUFF.4199.1 and Vcnovel_50_3p to CUFF.9347.1 (Fig. S3).

### Different expression of miRNAs during fruit maturation

Comparisons were performed between three mature phases. As a result, the differentially expressed miRNAs were more abundant in pink/green (41) than in blue/pink (15). In pink/green, 32 miRNAs targeted 148 unigenes, while 11 miRNAs targeted 14 unigenes in blue/pink, and 34 miRNAs targeted 109 unigenes in blue/green. In pink/green, the target unigenes were involved in pathways e.g. phenylpropanoid biosynthesis. In blue/pink, the target unigenes were also involved in various pathways, e.g. plant hormone signal transduction. Here, several modules of miRNA-targets were specifically involved in anthocyanin biosynthesis, i.e. miR156-SPLs, miR319-MYBs and miR393-TIR1/AFB, which are worthy of closer inspection.

#### miR156-SPLs and miR319-MYBs role in anthocyanin biosynthesis

**Figure 2:**
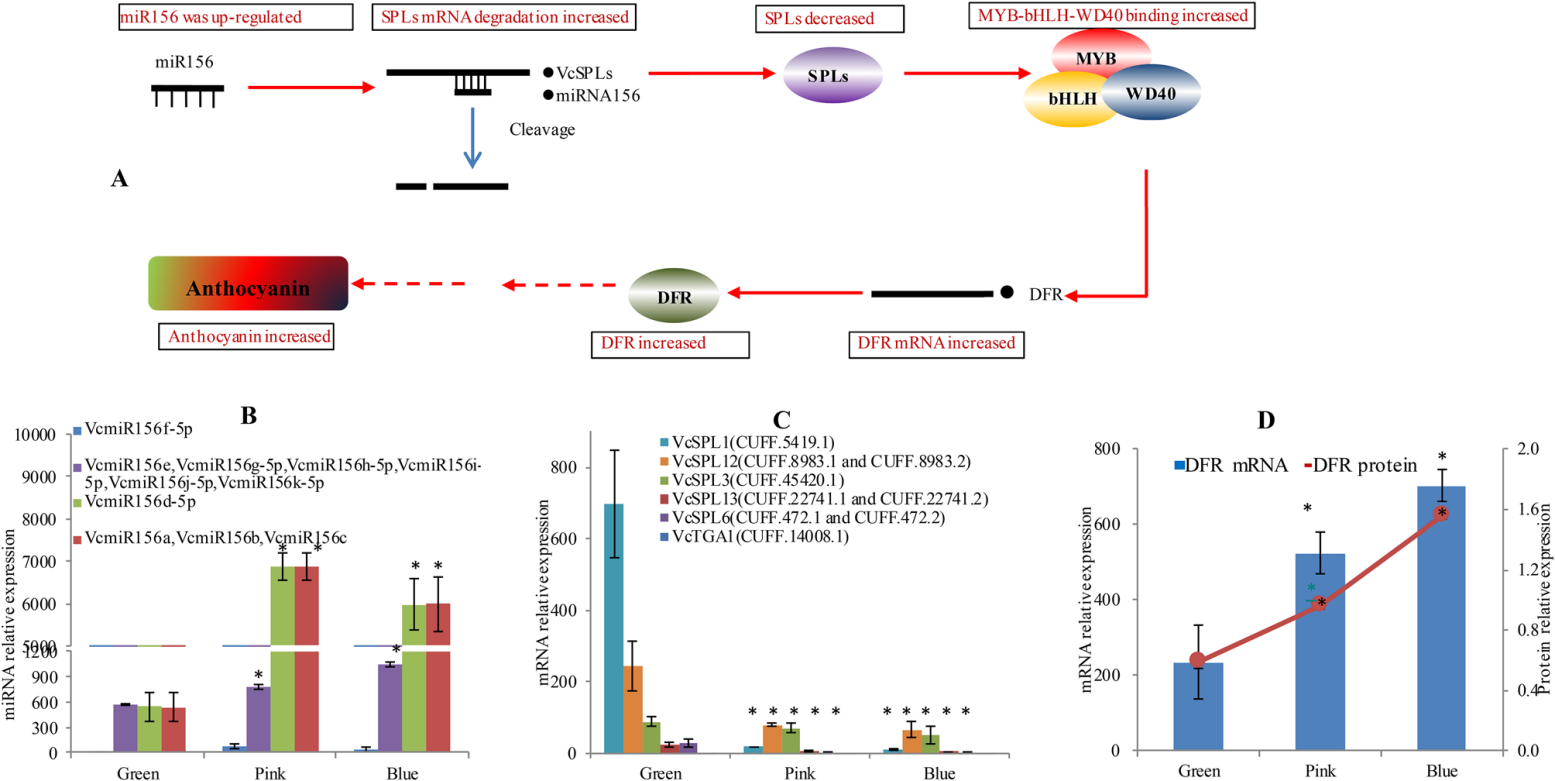
A potential pathway of the miR156-SPL module involved in anthocyanin biosynthesis during fruit maturation. **(A)** VcmiR156-VcSPLs regulated dihydroflavonol 4-reductase (DFR), a gene responsible for anthocyanin biosynthesis by down-regulating SPLs and disturbing a MYB-bHLH-WD40 transcriptional activation complex; the relative expression of **(B)** miRNA156 and **(C)** their target SPLs at the RNA level; **(D)** the relative expression of DFR at both RNA and protein level. Error bars represented standard errors from three biological replicates (ten fruit as one replicate). "*" indicates the differences between green, pink and blue phases are significant (T test; *:P < 0.01).

**Figure 3:**
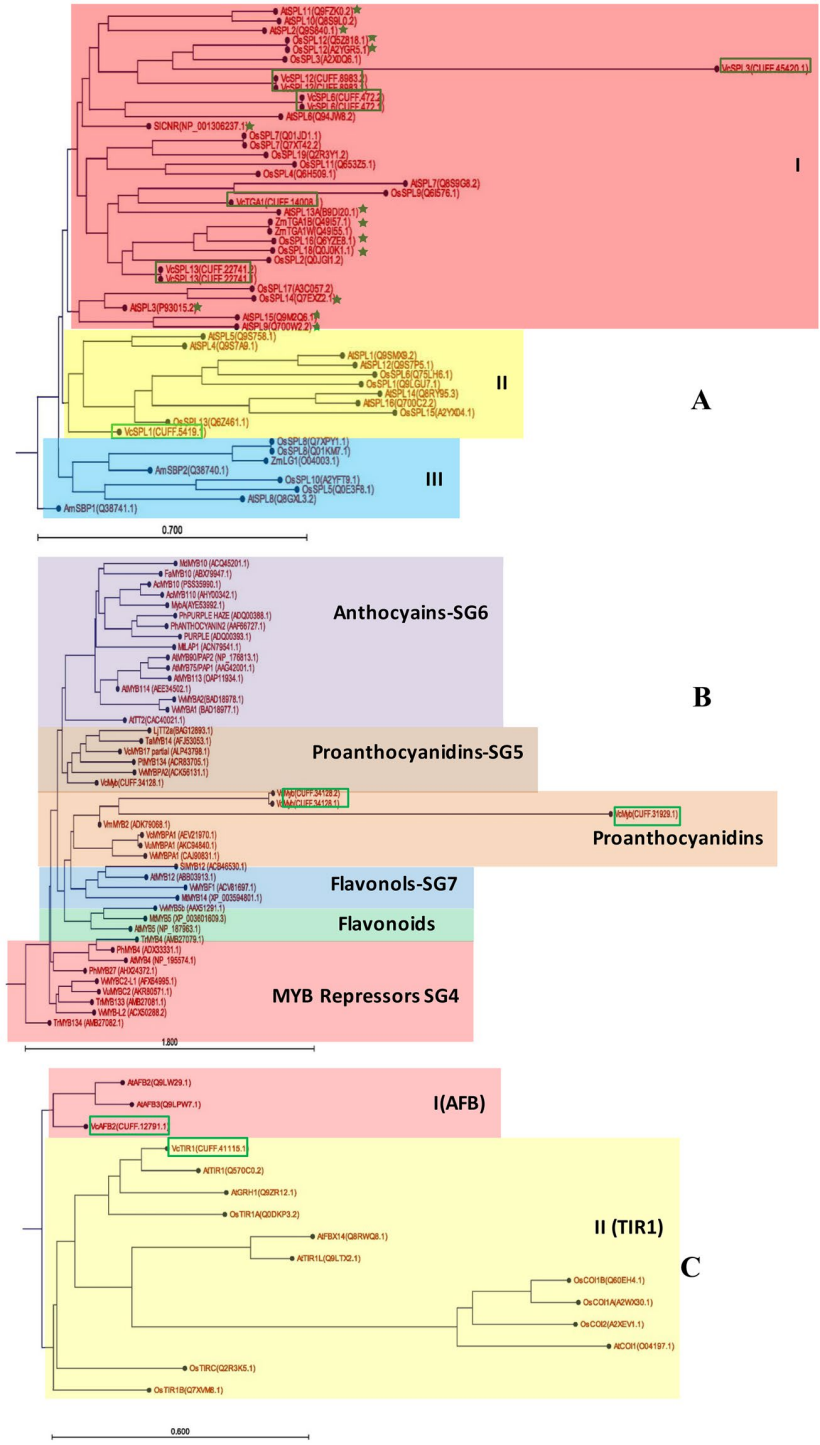
Phylogenetic tree of SPLs **(A)**, TIR1/AFBs **(B)** and MYBs **(C)** based on their deduced proteins. The SPLs were divided into group I in red, group II in yellow, and group III in blue. VcSPL in blueberry were branched into group IV and included a number of representative SPLs. The corresponding SPL genes in clades with asterisks were regulated by miRNA156, e.g. OsSPL2, OsSPL12, OsSPL13, OsSPL14, OsSPL16, and OsSPL18 in rice^39^, ZmTGA1 in maize^40^, AtSPL3 in Arabidopsis^41^, AtSPL2, AtSPL3, AtSPL9, AtSPL10, AtSPL11, AtSPL13A, AtSPL15^42^, and SlCNR in tomato^22^; TIR1/AFBs were divided into group I (AFB) in red, and group II (TIR1) in yellow. VcTIR1 (CUFF.41115.1), and VcAFB2 (CUFF.12791.1) were categorized into the two independent branches. VcMYBs unigenes (CUFF.34128.1, CUFF.34128.2, and CUFF.31929.1) were categorized with the published flavonoid-related MYBs from Vaccinium, VcMYBPA1, VmMYBPA1, and VuMYBPA1, that have the SG5 characteristic residues in the MYB domain^18^. At, *Arabidopsis thaliana*; Am, *Antirrhinum majus*; Os, *Oryza sativa*; Sl, *Solanum lycopersicum*; Vc, *Vaccinium myrtillus*; Vv, *Vitis vinifera*; Vu, *Vaccinium uliginosum*; Zm, *Zea mays*. Proteins shown in the green rectangle were VcSPLs and VcTIR1/AFBs in blueberry.

**Figure 4:**
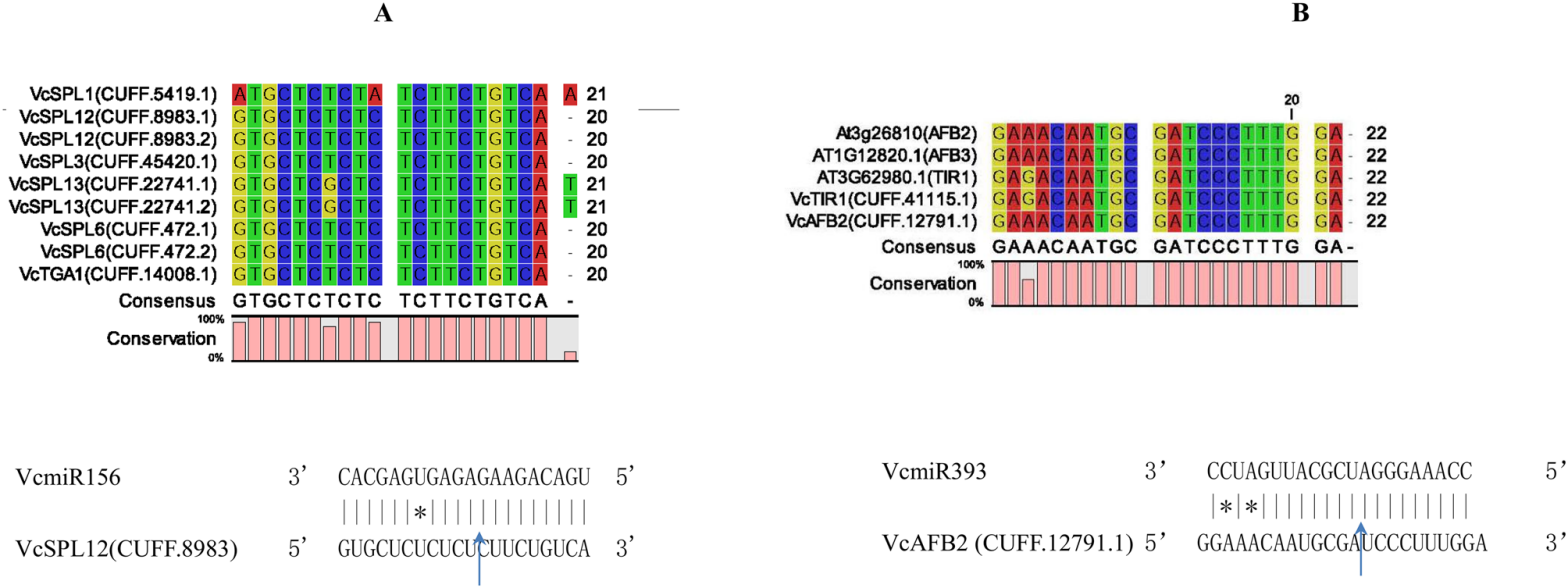
Cleavage sites of **(A)** VcSPLs targeted by miR156 and **(B)** VcTIR1 by VcmiR393. The cleavage sites were validated by RLM-5’RACE.

Many SPL genes are targeted by miR156, which plays important roles in anthocyanin biosynthesis (Fig. 2A). Here, SPL-miRNA156 were predicted and found to be involved with 10 members of SPL/SBP and 11 members of miRNA156s. These miRNA156 and SPL unigenes showed a dynamic pattern of expression. Except for VcmiR156f.-5p, six members of miR156 (VcmiR156e, VcmiR156g-5p, VcmiR156h-5p, VcmiR156i-5p, VcmiR156j-5p, and VcmiR156k-5p) were gradually up-regulated from green, pink to blue phases, while another four members (VcmiR156a, VcmiR156b, VcmiR156c, and VcmiR156d-5p) were dramatically up-regulated from green to pink phases, and then slightly down-regulated from pink to blue phases (Fig. 2B). In contrast, except for VcTGA1 (CUFF.14008.1), seven SPL unigenes, VcSPL13 (CUFF.22741.2), VcSPL13 (CUFF.22741.1), VcSPL6 (CUFF.472.1 and CUFF.472.2), VcTGA1X2 (CUFF.45420.1), VcSPL12 (CUFF.8983.1 and CUFF.8983.2), were down-regulated as the fruit matured (Fig. 2C). Their regulation trends were consistent to the result of qPCR (Fig. S4A, B). Phylogenetic analysis showed that these VcSPL were mainly categorized into a special group including a number of representative SPLs targeted by miRNA156, e.g. ZmTGA1, and AtSPL3/AtSPL9 (Fig. 3A). The cleavage was confirmed by RLM-Race (Fig. 4A). Taken together, it suggests that the up-regulation of the members of miRNA156 probably led to the down-regulation of VcSPLs.

A pathway has been proposed in which miR156-SPL regulates dihydroflavonol 4-reductase (DFR), a gene responsible for anthocyanin biosynthesis, by disturbing a MYB-bHLH-WD40 transcriptional activation complex^6^ (Fig. 2A). Here, the expression of DFR was investigated for its response to SPL. Interestingly, expressions of VcDFR (CUFF.50634) mRNA and protein were both up-regulated during fruit maturation, which was negatively correlated with expression of SPLs and positively with miRNA156 (Fig. 2). The up-regulation of VcDFR was paralleled with increases of anthocyanin (Fig. 1C). These results suggest miRNA156-SPL was probably involved in anthocyanin accumulation of blueberry fruit during maturation.

Three types of TFs, MYB, bHLH and WD40-repeat were targeted by the differentially expressed VcmiRNAs, which could potentially regulate structural genes of anthocyanin biosynthesis. VcbHLH (CUFF.50950.1) and VcWD40 (CUFF.52615.1) were targeted by Vcnovel_140_5p, and Vcnovel_93_5p respectively. The R2R3 MYB is the most abundant class of MYB genes in plants, regulating biosynthesis of anthocyanin and other flavonoids. VcMYBs unigenes (CUFF.34128.1, CUFF.34128.2, and CUFF.31929.1) were targeted by miR319. Phylogenetic analysis was also performed on the blueberry MYB unigenes against other species characterized MYB sequences related to flavonoids (Fig. 3B). The results suggest that VcMYBs unigenes (CUFF.34128.1, CUFF.34128.2, and CUFF.31929.1) were categorized with the published flavonoid-related MYBs from Vacciniums, VcMYBPA1, VmMYBPA1, and VuMYBPA1, that have the SG5 characteristic residues in the MYB domain^18^.

#### miRNA in anthocyanin biosynthesis responding to IAA and ABA

**Figure 5:**
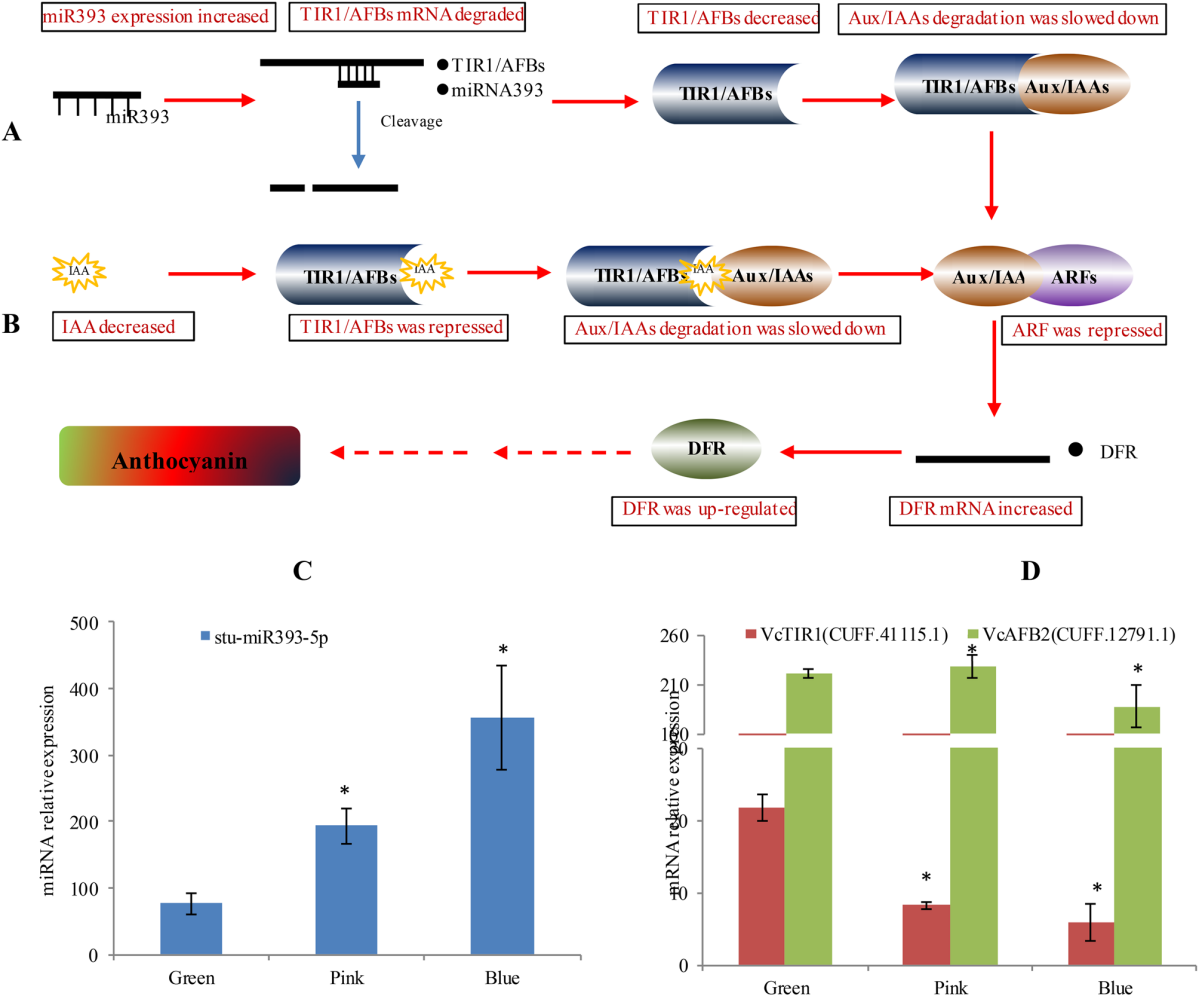
A schematic diagram of changes in auxin signal transmission during blueberry fruit maturation. **(A)** VcmiR393 was up-regulated, which down-regulated VcTIR1, slowed down degradation of Aux/IAAs and suppressed activity of ARFs **(B)** As auxin (IAA) gradually decreased during maturation, TIR1/AFBs were repressed accordingly, which also slow down degradation of Aux/IAAs. Thus, IAA and VcmiR393 had dual effects on suppressing ARFs to induce VcDFR expression and accelerated anthocyanin biosynthesis. **(C)** VcmiR393 was up-regulated **(D)** VcTIR1/AFBs were posttranscriptionally repressed. Error bars represented standard errors from three biological replicates (ten fruit as one replicate). "*" indicates the differences between green, pink and blue phases are significant (T test; *:P < 0.01).

Auxin signal is transduced through auxin receptors and mainly acts on transcriptional regulation by ARF and Aux/IAA protein families^19^. F-box proteins (TIR1/AFBs) are required for Aux/IAAs degradation, while Aux/IAAs negatively regulate ARF proteins (Fig. 5A,B). In order to explore the scenario of miRNA in the auxin signaling, IAA content was determined and showed a dramatic drop as blueberry fruit matured (Fig. 1B). The decrease of IAA could reduce active TIR1/ABFs, resulting in ARF repression through slowing down Aux/IAA degradation (Fig. 5). Additionally, two TIR1/AFBs were targeted by VcmiR393 and categorized into two independent branches (i.e. AFB2/AFB3, and TIRs) in a phylogenetic tree, named by VcTIR1 (CUFF.41115.1) and VcAFB2 (CUFF.12791.1) (Fig. 3C). During fruit maturation, VcmiR393 was up-regulated, while both TIR1/ABFs were down-regulated accordingly (Fig. 5C,D). Their regulation trends were consistent with the results from qPCR (Fig. S4B, D) and their cleavage site was confirmed by RLM-Race (Fig. 4B). Thus, IAA and VcmiR393 together inhibited auxin signal transduction by repressing ARFs through TIR1/ABF and AUX/IAAs. In apple, MdARF13 acts as a negative regulator of the anthocyanin biosynthesis via repressing MdDFR^20^. Here, the up-regulation of VcDFR could be also correlated with the repression of ARFs, which is due to the regulation of IAA and VcmiR393. Additionally, two VcmiRNA (VcmiR160a-5p and VcmiR160b) targeted four VcARFs (CUFF.1934.1, CUFF.1934.2, CUFF.30893.1, and CUFF.38764.1).

The plant hormone ABA is a major regulator of non-climacteric fruit ripening^21^. ABA perception and signal transduction can be performed by MYB. Here, these VcMYBs unigenes (CUFF.34128.1, CUFF.34128.2, and CUFF.31929.1) targeted by miR319 were predicated to be regulated in ABA-activated signaling pathways.

## Discussion

### The role of miR156-SPL and miR319-MYB in fruit maturation and anthocyanin biosynthesis

In plants, it is ubiquitous that miR156 targets a subset of SPLs in a serial processes of fruit development^7^. In blueberry, mi156-SPLs are also reported in a transition from white fruit to mature fruit^14^. Here, Five VcSPLs targeted by VcmiR156 were grouped together with the characterized SPLs targeted by miRNA156, e.g. SlCNR. In tomato, miR156-targeted SlySBP gene, CNR, acts as a crucial factor controlling fruit ripening in tomato^22^, especially for fruit softening after the red ripe stage^23^. In banana fruits, miR156a also targets SPL7 and SPL9 to regulate fruit ripening^24^. During fruit maturation, miR156 also positively regulates anthocyanin accumulation by suppressing a negative regulator, SPL9^6^. SPL9 competes with TT8 (a bHLH), for binding to PAP1 (a MYB), resulting in destabilization of a MYB-bHLH-WD40 transcriptional activation complex and thereby suppression of DFR expression in anthocyanin biosynthesis. In litchi fruit, miR156a-LcSPL is involved in the anthocyanin biosynthesis^25^. In pericarp of litchi, the expression of miR156a is in parallel with the accumulation of anthocyanins, as opposed to that of its targets LcSPL1/2. Here, VcDFR, was up-regulated at both levels of mRNA and protein during fruit maturation. Interestingly, the up-regulation of VcDFR seemingly responded to the up-regulation of miRNA156 and the down-regulation of SPLs. These results suggest that the miRNA156-SPLs could be involved in anthocyanin accumulation during blueberry fruit maturation.

The complex of MYB, bHLH and WD40 plays a crucial role in controlling anthocyanin biosynthesis. MYB is usually the center of a complex, which is also subject to regulation of miRNA. Here, VcMYBs unigenes (CUFF.34128.1, CUFF.34128.2, and CUFF.31929.1) were targeted by miR319 and phylogenetically categorized with the published flavonoid-related MYBs from Vacciniums, i.e. VcMYBPA1, VmMYBPA1, and VuMYBPA1. These MYBs have the SG5 characteristic residues in the MYB domain^18^. In grape, VvMYBPA1 controls expression of PA pathway genes including both LAR and ANR during early berry development and in seeds^26^. In bilberry, the expression of VmMYBPA1 is positively correlated with anthocyanin accumulation during berry development^27^. In bog bilberry, VuMYBPA1 expression is much lower in white fleshed berries^28^. In blueberry, VcMYBPA1 has the ability to activate promoters of two PA genes, but not that of an anthocyanin specific gene^5^. The question on how VcMYBs unigenes (CUFF.34128.1, CUFF.34128.2, and CUFF.31929.1) effect anthocyanin accumulation needs to be addressed in future studies.

### Interaction between MiRNA and auxin or ABA in anthocyanin biosynthesis during fruit maturation

Anthocyanin biosynthesis is influenced by plant hormones. In carrot, 2,4-D strongly inhibits anthocyanin biosynthesis^29^. In tobacco calli, the application of exogenous naphthaleneacetic acid (NAA) and 2,4-D decreases the anthocyanin content^30^. In apple, increasing concentrations of auxin significantly inhibits anthocyanin biosynthesis^31^, through the MdARF13-mediated auxin signaling pathway^20^. In bilberry, exogenous ABA significantly promotes anthocyanin biosynthesis and accumulation in fruits attached and detached to the plant^21^.

Auxin response is carried out by two large protein families, i.e. the auxin response factor proteins (ARF) and the Aux/IAA proteins (Aux/IAAs)^32^. ARFs bind a DNA element (AuxRE) and activate transcription (or repress transcription in some case)^33^, while ARFs are negatively regulated by the Aux/IAAs that act as transcription repressors by binding to “activating ARFs”^5^. Aux/IAAs degradation requires a kind of F-Box proteins i.e. TIR1, AFB1/2/3. These F box proteins act as auxin receptors. When auxin present, it allows TIR1/AFBs to bind to their targets, i.e. Aux/IAAs, for degradation via the ubiquitin degradation pathway^34^. Plants with all TIR1 and AFB1/2/3 deficient proteins are auxin insensitive and exhibit a severe embryonic phenotype^34^. Here, as IAA decreased, the opportunity of TIR1/AFB to interact with Aux/IAAs was reduced, but the chance of Aux/IAAs binding to AFR was increased. Thus, activity of AFR was suppressed (Fig. 5).

TIR1/AFB are also regulated by miR393, which is a conserved family across many plants^35^. In Arabidopsis, MIR393a and MIR393b responding to auxin differently regulate TIR1 transcription^35^. The TIR1/AFBs is a component of the Skp1-Cullin1-F-box protein (SCF) ubiquitin ligase complexes to regulate auxin response by proteolysis of auxin/indole-3-acetic acids (Aux/IAA) repressors, and thereby release the activities of ARFs^34^. Repression of miR393 increases auxin sensitivity, influencing plant development including inhibition of primary root growth, overproduction of lateral roots, altered leaf phenotype and delayed flowering^36^. In apple, MdARF13 acts as a negative regulator of the anthocyanin biosynthesis via repressing MdDFR^20^. In this study, VcARFs (CUFF.1934.1, CUFF.1934.2, CUFF.30893.1, and CUFF.38764.1) were targeted by miR160. Also, VcARFs was regulated by miRNA-mediated Aux/IAA–ARF signaling pathways. The up-regulation of VcmiR393 coincided with the down-regulation of VcAFB2 (CUFF.12791.1). Thus, ARFs were negatively regulated by miRNA160 and VcmiR393-mediated Aux/IAAs together.

ABA, is a major regulator of non-climacteric fruit (e.g. strawberry, blueberry and grape.) ripening^21^. ABA signaling can be transduced to regulate ABA-responsive genes by TFs, e.g. MADS, MYB, and bZIP. In strawberry, R2R3-MYB10 can be activated by ABA, which effects many genes in the anthocyanin biosynthetic pathway during fruit ripening^37^. In strawberry, FaGAMYB mediates interactions between gibberellin and ABA during receptacle ripening^38^. In bilberry, VmSCL8, VmMADS18, VmMADS9, VmSHP, and VmBL are potentially TFs and their expression are positively regulated by ABA^21^. Here, VcMYBs unigenes (CUFF.34128.1, CUFF.34128.2, and CUFF.31929.1) targeted by miR319 were predicted to positively regulate the ABA-activated signaling pathway.

In conclusion, a number of sRNA and their target genes were identified to build the modules responsible for anthocyanin accumulation during fruit maturation. Notably, two modules of VcmiRNA156-VcSPLs and VcmiR393-VcTIR1/AFBs were confirmed, which were associated to the “VcDFR” gene. VcDFR is an important enzyme for biosynthesis of the final products, “anthocyanins”. Additionally, VcMYBs in anthocyanin biosynthesis might be potentially regulated by VcmiRNA319 and ABA together. These findings will facilitate future study of miRNA-mediated mechanisms underlying anthocyanin biosynthesis during blueberry fruit maturation.

## Supplementary Information

Supplementary Information.

## References

[1] Cho E, Seddon JM, Rosner B, Willett WC, Hankinson SE (2004). Prospective study of intake of fruits, vegetables, vitamins, and carotenoids and risk of age-related maculopathy. Arch. Ophthalmol..

[2] Hou DX (2003). Potential mechanisms of cancer chemoprevention by anthocyanins. Curr. Mol. Med..

[3] Del Rio D (2013). Dietary (poly)phenolics in human health: Structures, bioavailability, and evidence of protective effects against chronic diseases. Antioxid. Redox Signal..

[4] Li X, Jin L, Pan X, Yang L, Guo W (2019). Proteins expression and metabolite profile insight into phenolic biosynthesis during highbush blueberry fruit maturation. Food Chem..

[5] Zifkin M (2012). Gene expression and metabolite profiling of developing highbush blueberry fruit indicates transcriptional regulation of flavonoid metabolism and activation of abscisic acid metabolism. Plant Physiol..

[6] Gou JY, Felippes FF, Liu CJ, Weigel D, Wang JW (2011). Negative regulation of anthocyanin biosynthesis in Arabidopsis by a miR156-targeted SPL transcription factor. Plant Cell.

[7] Li X (2018). Next-generation sequencing sheds new light on small RNAs in plant reproductive development. Curr. Issues Mol. Biol..

[8] Luo Q, Mittal A, Jia F, Christopher DR (2012). An autoregulatory feedback loop involving PAP1 and TAS4 in response to sugars in Arabidopsis. Plant Mol. Biol..

[9] Tirumalai V, Swetha C, Nair A, Pandit A, Shivaprasad PV (2019). miR828 and miR858 regulate VvMYB114 to promote anthocyanin and flavonol accumulation in grapes. J. Exp. Bot..

[10] Jia X (2015). Small tandem target mimic-mediated blockage of microRNA858 induces anthocyanin accumulation in tomato. Planta.

[11] Gupta V (2015). RNA-Seq analysis and annotation of a draft blueberry genome assembly identifies candidate genes involved in fruit ripening, biosynthesis of bioactive compounds, and stage-specific alternative splicing. GigaScience.

[12] Colle, M. *et al.* Haplotype-phased genome and evolution of phytonutrient pathways of tetraploid blueberry. *GigaScience* **8**. 10.1093/gigascience/giz012 (2019). 10.1093/gigascience/giz012 PMC6423372 30715294

[13] Yue, J. *et al.* Identification of conserved and novel microRNAs in blueberry. *Front. Plant Sci.* **8**, 1155 (2017). 10.3389/fpls.2017.01155 PMC5492659 28713413

[14] Hou Y (2017). Comparative analysis of fruit ripening-related miRNAs and their targets in blueberry using small RNA and degradome sequencing. Int. J. Mol. Sci..

[15] Kanehisa M, Goto S (2000). KEGG: Kyoto encyclopedia of genes and genomes. Nucleic Acids Res..

[16] Wang L, Feng Z, Wang X, Wang X, Zhang X (2010). DEGseq: An R package for identifying differentially expressed genes from RNA-seq data. Bioinformatics.

[17] Li X (2015). Characterization and comparative profiling of the small RNA transcriptomes in two phases of flowering in *Cymbidium ensifolium*. BMC Genomics.

[18] Plunkett BJ (2018). MYBA from blueberry (Vaccinium section Cyanococcus) is a subgroup 6 type R2R3MYB transcription factor that activates anthocyanin production. Front Plant Sci..

[19] Ohta K, Kanahama K, Kanayama Y (2005). Enhanced expression of a novel dioxygenase during the early developmental stage of tomato fruit. J. Plant Physiol..

[20] Wang, Y. C. *et al.* Auxin regulates anthocyanin biosynthesis through the Aux/IAA-ARF signaling pathway in apple. *Hortic. Res.-Engl.* **5** (2018). 10.1038/s41438-018-0068-4 PMC6269505 30534386

[21] Karppinen, K., Tegelberg, P., Haggman, H. & Jaakola, L. Abscisic acid regulates anthocyanin biosynthesis and gene expression associated with cell wall modification in ripening bilberry (*Vaccinium myrtillus* L.) fruits. *Front. Plant Sci.* **9**, 1259. 10.3389/fpls.2018.01259 (2018). 10.3389/fpls.2018.01259 PMC6124387 30210522

[22] Ferreira e Silva, G. F. *et al.* microRNA156-targeted SPL/SBP box transcription factors regulate tomato ovary and fruit development. *Plant J. Cell Mol. Biol.* **78**, 604–618. 10.1111/tpj.12493 (2014). 10.1111/tpj.12493 24580734

[23] Hwang I, Sakakibara H (2006). Cytokinin biosynthesis and perception. Physiol. Plant..

[24] Bi F, Meng X, Ma C, Yi G (2015). Identification of miRNAs involved in fruit ripening in Cavendish bananas by deep sequencing. BMC Genomics.

[25] Yao F, Zhu H, Yi C, Qu H, Jiang Y (2015). MicroRNAs and targets in senescent litchi fruit during ambient storage and post-cold storage shelf life. BMC Plant Biol..

[26] Bogs J, Jaffe FW, Takos AM, Walker AR, Robinson SP (2007). The grapevine transcription factor VvMYBPA1 regulates proanthocyanidin synthesis during fruit development. Plant Physiol..

[27] Jaakola L (2010). A SQUAMOSA MADS box gene involved in the regulation of anthocyanin accumulation in bilberry fruits. Plant Physiol..

[28] Primetta AK, Karppinen K, Riihinen KR, Jaakola L (2015). Metabolic and molecular analyses of white mutant Vaccinium berries show down-regulation of MYBPA1-type R2R3 MYB regulatory factor. Planta.

[29] Ozeki Y, Komamine A (1986). Effects of growth regulators on the induction of anthocyanin synthesis in carrot suspension cultures. Plant Cell Physiol..

[30] Zhou LL, Zeng HN, Shi MZ, Xie DY (2008). Development of tobacco callus cultures over expressing Arabidopsis PAP1/MYB75 transcription factor and characterization of anthocyanin biosynthesis. Planta.

[31] Ji, X. H. *et al.* Effect of auxin, cytokinin and nitrogen on anthocyanin biosynthesis in callus cultures of red-fleshed apple (*Malus sieversii f.niedzwetzkyana*). *Plant Cell Tissue Org.* **120**, 325–337 (2015).

[32] Yi W, Akoh CC, Fischer J, Krewer G (2006). Absorption of anthocyanins from blueberry extracts by caco-2 human intestinal cell monolayers. J. Agric. Food Chem..

[33] Wu X (2006). Concentrations of anthocyanins in common food in the United States and estimation of normal consumption. J. Agric. Food Chem..

[34] McGhie TK, Walton MC (2007). The bioavailability and absorption of anthocyanins: Towards a better understanding. Mol. Nutr. Food Res..

[35] Jones-Rhoades MW, Bartel DP (2004). Computational identification of plant microRNAs and their targets, including a stress-induced miRNA. Mol. Cell.

[36] Chen ZH (2011). Regulation of auxin response by miR393-targeted transport inhibitor response protein 1 is involved in normal development in Arabidopsis. Plant Mol. Biol..

[37] Medina-Puche L (2014). MYB10 plays a major role in the regulation of flavonoid/phenylpropanoid metabolism during ripening of *Fragaria x ananassa* fruits. J. Exp. Bot..

[38] Vallarino JG (2015). Central role of FaGAMYB in the transition of the strawberry receptacle from development to ripening. New Phytol..

[39] Xie K, Wu C, Xiong L (2006). Genomic organization, differential expression, and interaction of SQUAMOSA promoter-binding-like transcription factors and microRNA156 in rice. Plant Physiol..

[40] Chuck G, Cigan AM, Saeteurn K, Hake S (2007). The heterochronic maize mutant Corngrass1 results from overexpression of a tandem microRNA. Nat. Genet..

[41] Wu G, Poethig RS (2006). Temporal regulation of shoot development in *Arabidopsis thaliana* by miR156 and its target SPL3. Development.

[42] Wang KL, Li H, Ecker JR (2002). Ethylene biosynthesis and signaling networks. Plant Cell.

