# Supplementary material for: Dynamic regulation of small RNAs in anthocyanin accumulation during blueberry fruit maturation
Source: Sci Rep. 2021 Jul 23;11:15080. doi: 10.1038/s41598-021-93141-8 (PMC8302573; doi:10.1038/s41598-021-93141-8)
Supplement: Supplementary file 1 — Supplementary Information. [file 41598_2021_93141_MOESM1_ESM.docx]

**Dynamic Regulation of Small RNAs in Anthocyanin Accumulation during Blueberry Fruit Maturation**

Xiaobai Li ^1^*, Yan Hong ^2^, Aaron Jackson ^3^, Fangqi Guo^1^

^1^ Zhejiang Academy of Agricultural Sciences, Hangzhou 310021, China1; hufanfan1982815@outlook.com

^2^ Zhejiang Sci–Tech University, Hangzhou 310018, China; hy@zstu.edu.cn

^3^ South Oak, Stuttgart, AR 72160, USA; osativadna@gmail.com

***** Correspondence: hufanfan1982815@outlook.com;lixiaobai@mail.zaas.ac.cn.

| Table S1 The sequences of specific stem-loop RT and qRT-PCR primers for miRNAs. | |  |  |
| --- | --- | --- | --- |
| Small RNA | RT primer(5'-3') | Forward primer(5'-3') | Universal reverse Primer |
| VcmiR156 | GTCGTATCCAGTGCAGGGTCCGAGGTATTCGCACTGGATACGACGTGCTC | GGGGTGACAGAAGAGAGTGA | CAGTGCAGGGTCCGAGGTATT |
| VcmiR393 | GTCGTATCCAGTGCAGGGTCCGAGGTATTCGCACTGGATACGACGGATCA | GGTCCAAAGGGATCGCATTG | CAGTGCAGGGTCCGAGGTATT |
| Vc-U6 | GTGCAGGGTCCGAGGTTTTGGACCATTTCTCGAT | GGAACGATACAGAGAAGATTAGCA | GTGCAGGGTCCGAGGT |

| Table S2 Primer of RLM-race specific to SPLs and TIR1 | | |
| --- | --- | --- |
| Unigene | Outer primer | Inner primer |
| VcSPL12 (CUFF.8983.1 and CUFF.8983.2) | GCCATCAAAAGCACAAACTG | CGTTCTCATAGGGTGCTTTG |
| VcAFB2 (CUFF.12791.1) | GCCCAACCAGTGTCCGATAC | TGTCTATCATCATCAGGGTT |

| Table S3 Primer sequences of SPLs and TIR1 for qRT-PCR. | |  |
| --- | --- | --- |
| Gene | Forward primer (5'-3') | Reverse Primer (5'-3') |
| VcSPL12(CUFF.8983) | ATCACGTGCCATTGGTATGC | GAGTCGTGTTGGCAAGGTTT |
| VcAFB2 (CUFF.12791.1) | GTTGAGAGTTTTCCCATCGG | TATTACGGGGTTGAGGGTGCA |
| actin | ATGGCATTGTCAGCAACTGGG | TTCTCTGTTAGCCTTGGGGTTG |

| Table S4 The category of all small RNA in nine libraries from fruit at three phases | | | | | | | | | | | | | | | | | | |
| --- | --- | --- | --- | --- | --- | --- | --- | --- | --- | --- | --- | --- | --- | --- | --- | --- | --- | --- |
| Types | Green1 | | Green2 | | Green3 | | PinK1 | | PinK2 | | PinK3 | | Blue1 | | Blue2 | | Blue3 | |
|  | Reads | precent | Reads | precent | Reads | precent | Reads | precent | Reads | precent | Reads | precent | Reads | precent | Reads | precent | Reads | precent |
| total | 10434089 | 100% | 8912393 | 100% | 6966246 | 100% | 5203224 | 100% | 8987892 | 100% | 11278326 | 100% | 5876593 | 100% | 4960023 | 100% | 5086341 | 100% |
| conserved miRNA | 56195 | 0.54% | 53728 | 0.60% | 32354 | 0.46% | 24628 | 0.47% | 40959 | 0.46% | 54042 | 0.48% | 47661 | 0.81% | 38298 | 0.77% | 32612 | 0.64% |
| rRNA | 3813489 | 36.55% | 1582086 | 17.75% | 2865875 | 41.14% | 2594162 | 49.86% | 3363707 | 37.42% | 4081460 | 36.19% | 2822040 | 48.02% | 2323011 | 46.83% | 2523501 | 49.61% |
| tRNA | 57347 | 0.55% | 69517 | 0.78% | 48079 | 0.69% | 47192 | 0.91% | 272277 | 3.03% | 331258 | 2.94% | 56335 | 0.96% | 90906 | 1.83% | 125193 | 2.46% |
| snRNA | 7505 | 0.07% | 6985 | 0.08% | 4920 | 0.07% | 4028 | 0.08% | 7212 | 0.08% | 9062 | 0.08% | 3608 | 0.06% | 3283 | 0.07% | 3165 | 0.06% |
| snoRNA | 163 | 0.00% | 140 | 0.00% | 104 | 0.00% | 80 | 0.00% | 107 | 0.00% | 141 | 0.00% | 76 | 0.00% | 49 | 0.00% | 59 | 0.00% |
| NAT_siRNA | 18879 | 0.18% | 20071 | 0.23% | 12216 | 0.18% | 7729 | 0.15% | 14833 | 0.17% | 19166 | 0.17% | 9051 | 0.15% | 7904 | 0.16% | 7237 | 0.14% |
| exon | 1237880 | 11.86% | 1306939 | 14.66% | 772406 | 11.09% | 509803 | 9.80% | 987948 | 10.99% | 1277747 | 11.33% | 682589 | 11.62% | 543421 | 10.96% | 498587 | 9.80% |
| intron | 841339 | 8.06% | 951370 | 10.67% | 530711 | 7.62% | 349314 | 6.71% | 730432 | 8.13% | 939454 | 8.33% | 384483 | 6.54% | 333385 | 6.72% | 305361 | 6.00% |
| novel_miRNA | 6736 | 0.06% | 6918 | 0.08% | 4302 | 0.06% | 3338 | 0.06% | 6020 | 0.07% | 7315 | 0.06% | 3564 | 0.06% | 3472 | 0.07% | 3174 | 0.06% |
| ta_siRNA | 20 | 0.00% | 26 | 0.00% | 20 | 0.00% | 3 | 0.00% | 13 | 0.00% | 11 | 0.00% | 21 | 0.00% | 15 | 0.00% | 7 | 0.00% |
| other | 4394536 | 42.12% | 4914613 | 55.14% | 2695259 | 38.69% | 1662947 | 31.96% | 3564384 | 39.66% | 4558670 | 40.42% | 1867165 | 31.77% | 1616279 | 32.59% | 1587445 | 31.21% |

| Table S5 Summary of conserved and novel miRNA in blueberry fruit | | | | | | | | | | | |
| --- | --- | --- | --- | --- | --- | --- | --- | --- | --- | --- | --- |
|  | Types | Total | Green1 | Green2 | Green3 | Pink1 | Pink2 | Pink3 | Blue1 | Blue2 | Blue3 |
| Conserved miRNA | Mapped mature | 68 | 58 | 55 | 55 | 59 | 53 | 47 | 57 | 54 | 56 |
|  | Mapped hairpin | 60 | 53 | 51 | 53 | 55 | 49 | 43 | 55 | 51 | 54 |
|  | Mapped uniq sRNA | 710 | 84 | 83 | 69 | 75 | 83 | 83 | 85 | 75 | 73 |
|  | Mapped total sRNA | 380259 | 56194 | 53723 | 32353 | 24607 | 40914 | 53968 | 47628 | 38272 | 32600 |
| Novel miRNA | Mapped mature | 161 | 146 | 148 | 129 | 113 | 134 | 134 | 117 | 113 | 113 |
|  | Mapped hairpin | 107 | 103 | 104 | 104 | 97 | 103 | 106 | 99 | 96 | 99 |
|  | Mapped uniq sRNA | 5212 | 755 | 873 | 537 | 450 | 687 | 745 | 422 | 397 | 346 |
|  | Mapped total sRNA | 22864 | 3707 | 4158 | 2150 | 1409 | 3119 | 3908 | 1752 | 1466 | 1195 |

| Table S6 Potential targets of miRNA in blueberry fruit | | | |
| --- | --- | --- | --- |
| Type | miRNA | Target sites | Target unigenes |
| Conserved | 64 | 984 | 333 |
| Novel | 334 | 30457 | 10802 |
| Total | 398 | 11325 | 11028 |

Figure S1 The size distribution of reads from 18-40 nucleotides


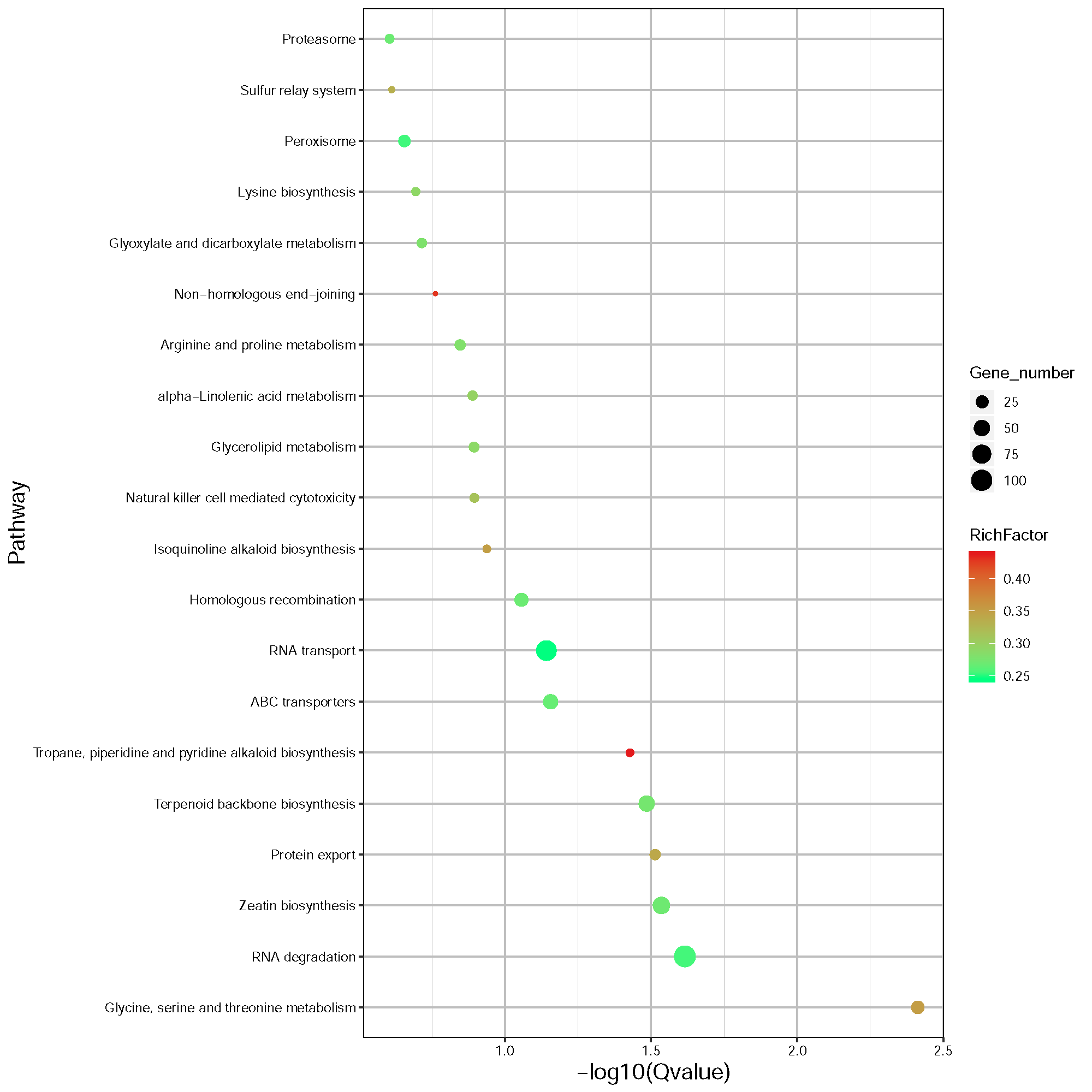


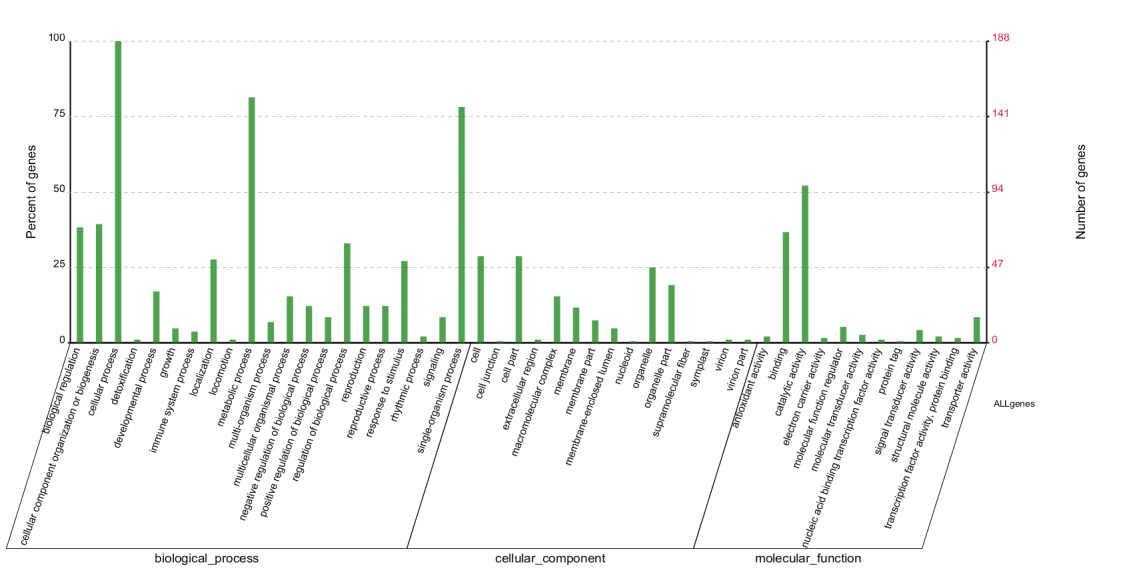


-long10(P-value)

**A** **B**

Figure S2 Summary of miRNA potential targets and their functional analysis **(a)** Go analysis **(b)** KEGG enrichment.

5' AUUCAAAAC-AAACGGAUUAAACC 3' CUFF.9347.1

o||||||| ||||oo||o|||||

3' CGAGUUUUGUUUUGUUUAGUUUGG 5' Vcnovel_50_3p

5' UGCGGGGGUAGGGGCGGAUGUA 3' CUFF.4199.1

|| o||| ||o|||o| ||o|

3' UCGGUCCCUUCUCCGUC-ACGU 5'VcmiR408b-3p


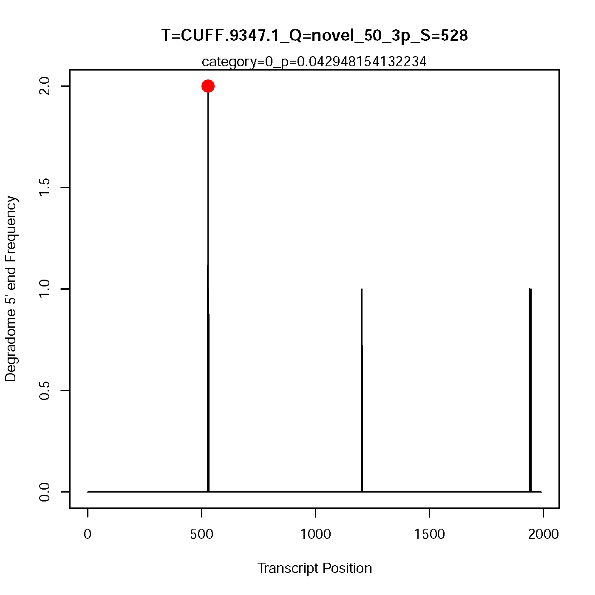

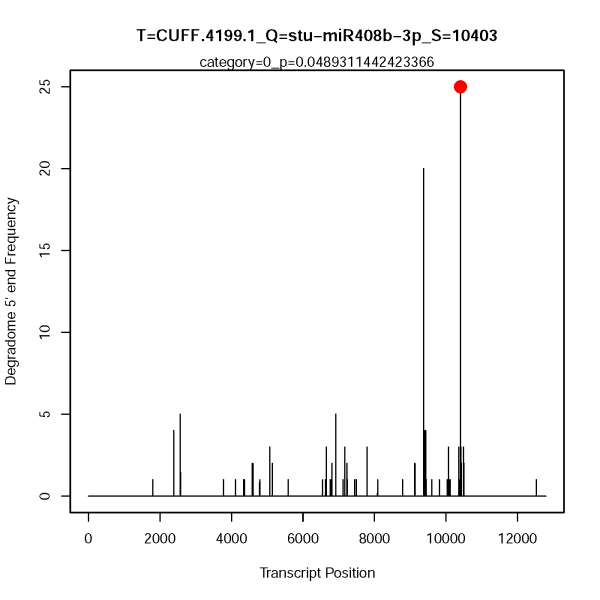


A **B**

Figure S3. The targets (CUFF.4199.1 and CUFF.9347.1) cleaved by the miR408b-3p and novel_50_3p respectively. **A** Cleavage features in VcFOG (CUFF.4199.1) mRNA by miR408b-3p from the degradome library; **B** Cleavage features in VcGAI1 (CUFF.9347.1) mRNA by novel_50_3p from the degradome library. The T-plots showed the abundance of the degradome tags along the target mRNA sequence. The line marked by red dot was the predicted cleavage site of the corresponding miRNAs.

**A B**

**C D**

Figure S4. The expression of two miRNA and their target genes quantified by qPCR. **A** VcmiR156 **B** VcSPL12 (CUFF.8983) **(c)** VcmiR393 **(d)** VcAFB2 (CUFF.12791.1).
